# Gaps in obesity management in the UAE and the role of bariatric endoscopy

**DOI:** 10.3389/fgstr.2023.1174640

**Published:** 2023-05-05

**Authors:** Maryam Alkhatry

**Affiliations:** Division of Gastroenterology & Endoscopy, Ibrahim Bin Hamad Obaidullah Hospital (IBHO), Emirates Health Services, Ras Al Khaimah, United Arab Emirates

**Keywords:** obesity, obesity management, unmet needs, bariatric endoscopy, challenges

## Abstract

The definitions of overweight and obesity include increased fat storage that might compromise one’s health. According to the World Health Organization (WHO), an adult is considered overweight if their Body Mass Index (BMI) is greater than or equal to 25, and obese if their BMI is greater than or equal to 30. Age must be taken into account when defining overweight and obesity in children. The prevalence of obesity and overweight was reported to be at 21% and 33%, respectively, in the Middle East region. As obesity incidence rises with ageing, those over 40 were found to have the highest prevalence of obesity and overweight. According to the Central Intelligence Agency’s World Factbook, the UAE has a prevalence rate of obesity of 31.7%, making it one of the top 20 nations in the world for high obesity rates in 2016. The prevalence of overweight and obesity in the UAE is estimated to have increased between 1989 and 2017 threefold.

## Introduction

Being a wealthy nation where the average income is high, nearly every kind of food, especially calorie- and fat-dense fast food, is widely available throughout the UAE. Emiratis may have adopted calorie- and fat-rich diets as part of their daily routines. (1) The Global Obesity Observatory’s data According to a 2019 analysis indicated that the economic cost of being overweight and obese was calculated at US$11.67 billion. This amounts to 2.8% of GDP and $1194 per person in the US. The percentages of direct and indirect expenses in total costs were 12% and 88%, respectively. Economic effects are expected to reach US$179.29 billion by 2060. This translates to a cost increase of 15.5 times, or US$16874 per person, or 11% of GDP. (2)

Several worldwide recommendations for the treatment of obesity are already publicly available. (3–6) In addition, in response to the high incidence of obesity in the UAE, AbuSnana et al. published consensus guidelines for dealing with overweight and obesity in which they recommended that Patients who have a BMI of less than 40 kg/m^2^ without any co-morbidities or less than 35 kg/m^2^ including one or more co-morbidities and for whom the operation would not be linked with a high risk should think about having bariatric surgery. (7)

Over the past ten years, bariatric surgery has gained popularity as a treatment approach for extreme obesity due to its effects on weight reduction, type 2 diabetes remission, and metabolic syndrome. In the UAE, where the prevalence of obesity is sharply rising, bariatric surgery has become more and more common. (8) However, even in an advanced healthcare system such as the one in the United States, less than 1% of those who fulfill the National Institute of Health’s BMI requirements for bariatric surgery actually have it. Emerging advancements in endoscopy-based treatment approaches are a result of the rising burden of surgical procedures, complications, and expenses. (9)

## Bariatric endoscopy: A new era

Medication and lifestyle changes have shown to only have a minor impact on weight loss. Although bariatric surgery has been demonstrated to be a groundbreaking approach in the treatment of obesity, many patients find this intrusive method intolerable owing to its side effects and probable long-term problems. Using state-of-the-art technology, bariatric endoscopy can replicate weight reduction surgery without the co-morbidities. In endoscopic bariatrics, a number of techniques are employed, each with a unique physiologic mechanism for weight loss. They include aspiration treatments, space-occupying devices, malabsorption strategies, and restricting practices. (10)

The development and usage of intragastrical balloons in the 1980s marked the beginning of the history of endoscopic bariatric treatment. Ever since, gastric and small bowel interventions have been the two main subgroups of endoscopic bariatric therapy. Gastric interventions function by activating mechanical and chemical receptors in the stomach, postponing gastric emptying, and adjusting the amounts of orexigenic hormones in the stomach. Bypassing the stomach, small bowel therapies alter satiety and gastrointestinal motility. (11)

## Bariatric endoscopy challenges

The management of postoperative problems and primary and revisional treatment options are all included in the constantly evolving field of bariatric endoscopy. Different products operate through various methods that may be tailored to the needs of each patient. While endoscopic therapy for obesity has shown promising effects, prospective randomized studies with prolonged follow-up are necessary to completely evaluate both primary and revisional endoscopic treatments. (12)

Over the past years, glucagon-like peptide-1 receptor agonists (GLP1-RA) have proven to be both safe and effective in the management of obesity. (13) The use of medications like GLP1- RAs in combination with endoscopic bariatric therapy is an emerging practice in obesity management that boosts weight loss provided by each therapeutic modality alone. (14) GLP1- RAs use is also gaining momentum to solve the weight regain challenge that faces many patients after bariatric endoscopic surgeries. Two-thirds of the rebound weight can be lost in patients using GLP1- RA without complications. (13)

We need to learn more about the intricate neuroendocrine pathways governing calorie intake and energy balance in order to comprehend the potential role of endoscopy in the treatment of obesity. Many variables, including nutrition levels in the blood, gut hormones, and brain signals from the GI tract, influence how hungry and full we feel. Other hormones that affect GI motility and modulate stomach emptying include cholecystokinin, peptide YY, and ghrelin. (15)

## Revisional bariatric endoscopy

Endoscopic bariatric procedures have made it possible to close the growing management gap in the obesity epidemic. Moreover, it has been claimed that they are used to regulate weight recidivism following Roux-en-Y gastric bypass. The use of gastric suturing to limit the volume of a dilated laparoscopic sleeve gastrectomy for the management of suboptimal weight loss or weight return seems intriguing, even if it may not result in as much weight reduction as its surgical equivalent for the main treatment of obesity. In comparison to more conventional surgical revision methods, the revisional endoscopic sleeve gastroplasty procedure has a number of benefits, including increased safety, technical simplicity, and organ-sparing properties that enable additional revisional surgery. (16)

Furthermore, Roux-en-Y gastric bypass patients have employed Endoscopic transoral outlet reduction (TORe) to control weight regain. Full-thickness suturing combined with argon plasma mucosal coagulation (ft-TORe) and argon plasma mucosal coagulation alone (APMC-TORe) are the two methods that are employed with the highest frequency. Both ft-TORe and APMC-TORe provide significant and equivalent weight-loss effects with a high and comparable safety profile, according to a meta study by Jaruvongvanich et al. However, APMC-TORe frequently required several endoscopic visits (14).

## The future of bariatric endoscopy

The patient’s financial burden is a major problem that restricts the use of future endoscopic bariatric therapies. Yet, little effort has been made to include them in this list despite the fact that several international associations have made great strides in making metabolic surgery an acknowledged treatment for people with obesity all around the world. These therapies will undoubtedly be included in the accepted list of treatments for this deadly disease as more information becomes available and technology advances. Besides, endoscopists and bariatric surgeons must complete a thorough training program before conducting these operations in order to develop the endoscopic abilities required. The European Society of Gastrointestinal Endoscopy (ESGE) has recently issued a set of recommendations on the training and development of endoscopists aiming to standardize and improve training in bariatric endoscopy. Recommendations include competency in upper gastrointestinal endoscopy, and a basic understanding of obesity, its pathophysiology, complications, and impact. The ESGE endorses the need for validated training using simulators followed by hands-on experience gained through training in tertiary centers. (17) The use of advanced endoscopy by surgeons and gastroenterologists has become more important as a result of ongoing technology advancements and growing public awareness of endoscopic bariatric interventions. (18) Advancements in this field include a new robotic device that performs uniform gastric suturing for all patients. Simple endoscopic skills are needed to perform this operation which further reduces the risk of complications. (19)

## Conclusion

Due to diverse demographics, the frequency of retrospective research, the absence of standard terminology, and the dearth of comparison studies, comparing various methodologies is challenging. As a result, developing a uniform therapy algorithm is challenging. Each therapy should be customized for each patient, taking into account the various factors that might ultimately have an impact on the result. (20)

There is a need to develop updated local guidelines to reflect the recent advances in the management of obesity and to serve as a guide to clinicians in the UAE.

## Author contributions

The author confirms being the sole contributor of this work and has approved it for publication.
